# Discovery of a novel mutation F184S (c.551T>C) in GATA4 gene causing congenital heart disease in a consanguineous Saudi family

**DOI:** 10.1016/j.heliyon.2024.e37177

**Published:** 2024-08-29

**Authors:** Mahmood Rasool, Peter Natesan Pushparaj, Absarul Haque, Ayat Mohammed Shorbaji, Loubna Siraj Mira, Sherin Bakhashab, Mohamed Nabil Alama, Muhammad Farooq, Sajjad Karim, Lars Allan Larsen

**Affiliations:** a Center of Excellence in Genomic Medicine Research, Department of Medical Laboratory Sciences, Faculty of Applied Medical Sciences, King Abdulaziz University, Jeddah, Saudi Arabia; b King Fahd Medical Research Center, Department of Medical Laboratory Sciences, Faculty of Applied Medical Sciences, King Abdulaziz University, Jeddah, Saudi Arabia; c Department of Biochemistry, King Abdulaziz University, Jeddah, Saudi Arabia; d Department of Cardiology, King Abdulaziz University Hospital, Jeddah, Saudi Arabia; e Department of Bioinformatics and Biotechnology, Government College University, Faisalabad, Pakistan; f Department of Cellular and Molecular Medicine, University of Copenhagen, Denmark

**Keywords:** GATA4, F184S, Congenital heart disease, Whole exome sequencing, Homology modelling

## Abstract

**Background & aim:**

Congenital heart disease (CHD) is the most common cause of non-infectious deaths in infants worldwide. However, the molecular mechanisms underlying CHD remain unclear. Approximately 30 % of the causes are believed to be genetic mutations and chromosomal abnormalities. In this study, we aimed to identify the genetic causes of CHD in consanguineous families.

**Methods:**

Fourth-generation pedigrees with CHD were recruited. The main cardiac features of the patient included absence of the right pulmonary artery and a large dilated left pulmonary artery. To determine the underlying genetic cause, whole-exome sequencing was performed and subsequently confirmed using Sanger sequencing and different online databases to study the pathogenesis of the identified gene mutation**.** An in-silico homology model was created using the Alphafold homology model structure of GATA4 (AF-P43694-F1). The missense3D online program was used to evaluate the structural alterations.

**Results:**

We identified a deleterious mutation c.551T > C (p.Phe184Ser) in *GATA4*. GATA4 is a highly conserved zinc-finger transcription factor, and its continuous expression is essential for cardiogenesis during embryogenesis. The in-silico model suggested a compromised binding efficiency with other proteins. Several variant interpretation algorithms indicated that the F184S missense variant in GATA4 is damaging, whereas HOPE analysis indicated the functional impairment of DNA binding of transcription factors and zinc-ion binding activities of GATA4.

**Conclusion:**

The variant identified in *GATA4* appears to cause recessive CHD in the family. In silico analysis suggested that this variant was damaging and caused multiple structural and functional aberrations. This study may support prenatal screening of the fetus in this family to prevent diseases in new generations.

## 1 Introduction

Congenital heart disease (CHD) is the most common birth defect that affects the heart and great vessels, accounts for approximately 1 % of all live births [1], and is responsible for approximately 29 % of non-infectious neonatal deaths. CHD is a multifactorial disease [2], and many environmental events lead to this disease, including maternal conditions such as diabetes mellitus, which results in elevated glycosylated hemoglobin levels and increases the risk 2–3 times higher than that in the normal population [3]. Autoimmune diseases, such as systemic lupus erythematosus and Sjogren syndrome, may also increase the risk of atrioventricular block [4]. Different types of infections also contribute to heart problems, such as varicella and rubella, which result in structural damage to the heart [5,6], and parvoviruses, which lead to heart failure secondary to myocarditis and severe anemia [7]; [8]. Exposure to different chemicals in maternity may also enhance the chances of CHD, and alcohol is one of the most prevalent agents. Other routine drugs, such as misoprostol and isotretinoin, and anticonvulsant agents, such as valproic acid, are associated with CHD [9].

In addition to environmental factors, approximately 30 % of CHD cases are caused by genetic factors (chromosomal abnormality or mutation in a single gene or multiple genes) [10]. The heart is one of the earliest developed organs during embryogenesis, and multiple molecular mechanisms and pathways are involved in its development and function [11]. Unfortunately, to date, the molecular mechanisms responsible for CHDs are largely unknown. About 142 genes have been identified so far in humans to be responsible for CHD (https://chdgene.victorchang.edu.au/). In mouse models, this number is much higher (>400) [12]. During heart development, aberrations in genes encoding transcription factors, chromatin modifiers, extracellular matrix proteins, and signalling pathway transducers may lead to anomalies in heart structure and function [12].

Transcription factors and cofactors seem to be crucial for proper structural development of the heart and its functions. The GATA family is a zinc finger transcription factor, and its role in heart development and cardiogenesis has been well established [13]. Pathogenic mutations in GATA4 have been reported to decrease transcriptional activity, leading to ventricular septal defects and bicuspid aortic valves [14]. Moreover, mutations in GATA4 regulatory genes, such as NEXN, also cause CHD [15]. Mouse knockout models for GATA4/6 fail to develop hearts and generate only second heart field (SHF) progenitor cells [16].

The primary aim of this study was to investigate the causative gene mutations in an extended family suffering from CHD and perform further analysis using in silico tools.

## 2 Materials & methods

### 2.1 Subjects recruitment

A four-generation family with CHD features was enrolled in this study at King Abdulaziz University Hospital (KAUH). The clinical features of the patient, physical examination results, and medical records were recorded. This study was approved by the Ethics Committee of the Center of Excellence in Genomic Medicine Research (07-CEGMR-Bioeth-2020). Written informed consent was obtained from all the participants prior to the start of the study. The patient was a male with heart problems. The patient showed an absence of the right pulmonary artery (RPA) and dilated left pulmonary artery (LPA), with multiple major aortopulmonary collateral arteries (MAPCAs) extending to the right lung, the largest originating from the subclavian artery. Unfortunately, the child died at 18 months of age due to cardiac complications.

### 2.2 Whole exome sequencing & Sanger sequencing

Blood samples were collected and immediately transported to the Center of Excellence in Genomic Medicine Research (CEGMR) for further molecular studies. DNA was extracted using a DNeasy® Blood & Tissue Kit (QIAGEN). The quantity and quality of the DNA were monitored using a Nanodrop™ 2000/2000c spectrophotometer (Thermo Fisher Scientific Waltham, Massachusetts, USA). After ensuring the quality and quantity of the DNA, we performed whole-exome sequencing using NovaSeq6000. In brief, the raw sequencing data folder was copied from Novaseq600 to Aziz-supercomputer for in-house NGS analysis. First, the base calls in the BCL files are converted to FASTQ format using a “bcl2fastq” followed by pre-processing FASTQ files consists of: (i) Removing adapters, (ii) Trimming low-quality bases “<20” and (iii) Filtering short reads” <20 pb”. The fastq files were aligned and mapped to the reference human genome reference *UCSC hg19* GRCh37 using the BWA mapping (version 0.7.12) application to generate a SAM file format that was converted to a BAM file, a compressed binary version of a SAM file. The ANNOVAR tool was used to annotate the variants with allele frequencies. Base quality score recalibration (BQSR) was performed before variant calling using GATK-Haplotype Caller in vcf file format, with approximately 40,000 variants in the WES data that could be reduced to 3000 after applying the following filtration criteria: (I) filter out the common variant among populations based on minor allele frequency (MAF 0.001). (II) Variant-based filtration [i) DP > 2; otherwise, it was classified as LowDP. MQ > 40.0; otherwise, it was classified as LowMQ, iii. FS < 60.00; otherwise, it was reported as a high FS, iv. QD > 2.0; otherwise, it was reported as LowQD v. QUAL >10.4139; otherwise, it was reported as LowQUAL]. The Variant Interpreter (illumine) tool was used for the annotation, interpretation, and detection of disease-associated variants. The pathogenicity of detected variants and disease-associated information were extracted from databases such as ClinVar, OMIM, Varsome, and PubMed. In addition to database resources, in silico prediction tools (MutationAssessor SIFT, PolyPhen, MutationTaster, PROVEAN, GeneSplicer, and Human Splicing Finder) were used to evaluate the pathogenicity of missense and splice site variants.

The pathogenic mutation in the gene was confirmed by ABI XL3500 using the Big Dye Terminator® with forward and reverse primers: forward primer sequence, 5-GGAAGCTGCGGCCTACAG-3 and reverse primer sequence, 5-AACAAGAGGCCCTCGACAG-3. BioEdit Sequence Alignment Editor Version 7.2.5 was employed to examine sequencing peaks and interpret the results.

### 2.3 GATA4 mutation analysis using missense 3D

To predict the structural impact of the missense mutation F184S, we used the Alphafold homology model structure of GATA4 (AF-P43694-F1) [17,18]. The missense3D online program (http://missense3d.bc.ic.ac.uk/missense3d/) was used to evaluate structural alterations in GATA4 due to the F184S mutation [19]; [20].

### 2.4 Structural analysis of wild type and mutated structures of GATA4

The SwissModel Structure Assessment Online program was used to examine the coordinates of wild-type and mutant GATA4 homology model structures. GATA4 structures were uploaded to the structural assessment and comparison tool to obtain structural details in terms of Ramachandran Plots [[21], [22], [23]], evaluation of model quality at both the global and local levels using MolProbity (Version 4.4) [24], and quality estimation using the QMEANDisCo method [25].

### 2.5 GATA4 mutation analysis using Mutation Taster, Polymorphism Phenotyping v2 and SIFT

The GATA4 mutation (F184S) was examined using the Mutation Taster online tool (https://www.genecascade.org/MutationTaster2021/#), based on a single-base exchange in the coding sequence c.551T > C using the Ensemble Transc*r*ipt ID ENST00000335135 [26]. Polymorphism Phenotyping v2 (PolyPhen-2) (http://genetics.bwh.harvard.edu/pph2/) and SIFT (https://sift.bii.a-star.edu.sg/) mutation analysis web tools were used to evaluate the impact of the F184S mutation on GATA4 [27]; [28].

### 2.6 GATA4 mutation analysis using HOPE

GATA4 F184S mutation analysis was performed using the HOPE online tool (https://www3.cmbi.umcn.nl/hope/input/) and the input supplied for each missense mutation [29]. The annotations for GATA4 were derived from the UniProt entry P43694 (https://www.uniprot.org/uniprot/P43694).

## 3 Results

**Fig. 1:**
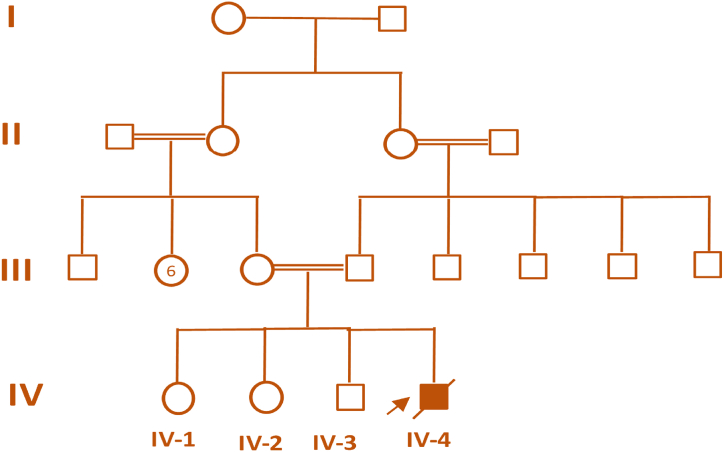
Pedigree of consanguineous family. The arrow indicates the proband (patient).

The patient with CHD in this family was from a consanguineous marriage, and most of the marriages that took place in this family were consanguineous. This may have led to the transfer of the mutated allele in the patient in the homozygous form, as shown in Fig. 1. The patient had an absence of the right pulmonary artery (RPA) and a dilated left pulmonary artery (LPA), with multiple major aortopulmonary collateral arteries (MAPCAs) extending to the right lung, the largest originating from the subclavian artery. Cardiac complications led to death of the child at 18 months of age.

### 3.1 Whole-exome sequencing and Sanger sequencing

**Fig. 2:**
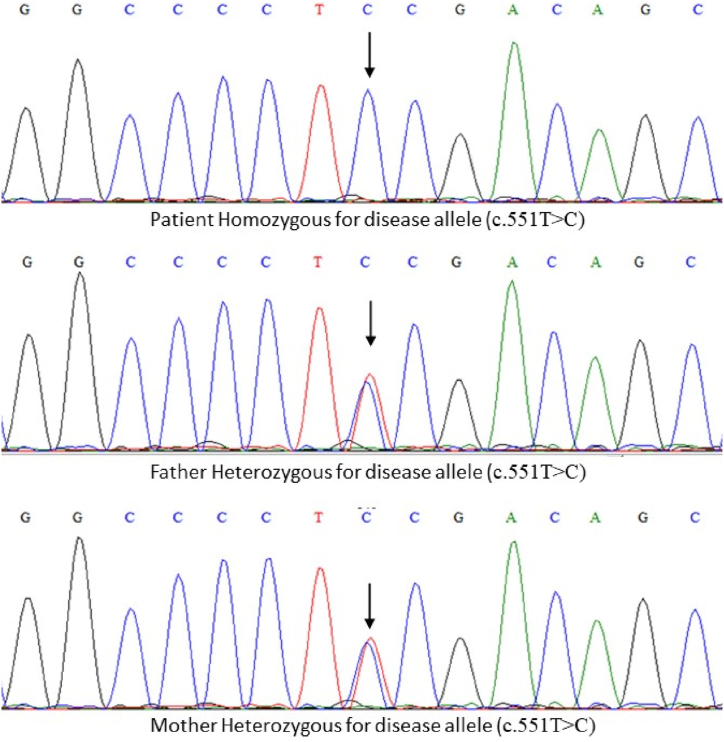
Shows chromatograms of the family. The patient was homozygous for the allele c.551T > C, while the parents were heterozygous.

Whole exome sequencing revealed many genes; however, upon applying different bioinformatics tools (SIFT, Polyphen, HOPE, Mutation Tester, etc.) and subsequent filtration, we found GATA4 as the main candidate gene, as the other genes were not involved in cardiogenesis and showed pathogenicity. In GATA4, thymine at nucleotide position 551 was substituted with cytosine (c.551T > C); consequently, the amino acid at position 184 was substituted from Phenylalanine to Serine at position 184 (p.Phe184Ser or p.F184S). Sorting Intolerant From Tolerant (SIFT) tool suggested a score of 0, which suggests deleterious, and Polymorphism Phenotyping (PolyPhen) suggested a score of 0.99, resulting in probable damage. Therefore, both tools predicted the mutation to be highly lethal to protein function and may cause disease. Other GATA4 mutations have also been reported in the literature to cause similar cardiac features. Therefore, Sanger sequencing was performed to confirm this mutation. This mutation was confirmed in the patient in a homozygous state. Both the parents were heterozygous for the mutated allele (Fig. 2).

**Fig. 3:**
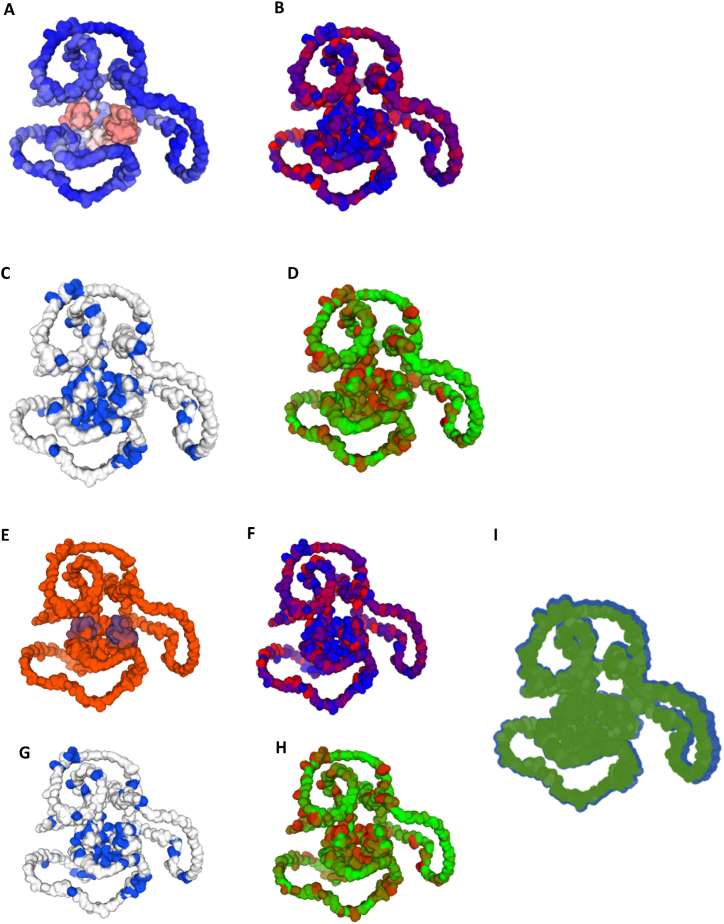
Wild-type (WT) and mutant (F184S) GATA-4 homology models based on the Swiss-Model server. The color schemes are depicted for the WT GATA-4 homology model based on (A) confidence (gradient), (B) hydrophobicity, (C) polarity, and (D) size of the amino acids. The color schemes are depicted for the mutant GATA-4 (F184S) homology model based on (E) confidence (gradient), (F) hydrophobicity (G) polarity, and (H) size of amino acids. (I) Superimposed structures of the WT and Mutant GATA-4 homology models. (For interpretation of the references to color in this figure legend, the reader is referred to the Web version of this article.)

We obtained a homology model for GATA4 from the Alphafold server (Fig. 3) to investigate the structural impact of missense mutations (F184S) on patients with heart disease. First, we examined the structural effects of the F184S missense mutation using a missense 3D tool. According to missense 3D study, missense mutation F184S did not induce structural damage in GATA4.

Structural assessment of mutant protein structures obtained from missense 3D analysis using the SwissModel Structure Assessment tool showed slight variations in the QMEAN Z scores compared with the wild-type GATA4 structure (Fig. 3A–D).However, the clash scores of the GATA4 F184S mutant and the WT homology models were 6.05 and 0.98, respectively. Importantly, the mutant GATA4 structure had more bad bonds (43/3206) than the wild-type GATA4 homology structure (0/3212) (Fig. 3E–H). However, no significant differences were observed between the wild-type GATA4 structure and the mutant structure (Fig. 3I) in terms of Ramachandran Plots, MolProbity Score, or other metrics studied in the SwissModel structure assessment tool.

The impact of the F184S missense mutation in GATA4 was further studied using online web tools such as PolyPhen-2 (score 0.979), SIFT (score 0.99), Mutation Taster (score 155), and HOPE (functional damage). The PolyPhen-2, SIFT, and Mutation Taster analyses indicated that the F184S missense mutation in GATA4 is probably damaging, whereas the HOPE analysis indicated the functional impairment of DNA binding of transcription factor and zinc-ion binding activities of GATA4 (Table 1).

**Table 1:** GATA4 missense mutation analysis using various cutting-edge online tools as indicated in table below.

Missense Mutation in GATA-4	Missense3D Prediction	SwissModel Structural Assessment						PoyPhen-2	SIFT	HOPE	Mutation Taster Score
		QMEAN	Cβ	Torsion	Solvation	All Atoms	Clash Score				
Wild Type	NA	−14.13	−0.46	−9.20	−16.95	−5.16	0.98	NA	NA	NA	NA
F184S	Structural Damage was not predicted based on Alphafold GATA-4 homology model	−14.07	−0.55	−9.21	−16.68	−5.21	6.05	0.979 (Sensitivity:0.75; Specificity: 0.96 Based on HumDiv) 0.755 (Sensitivity: 0.77; Specificity: 0.86 based on HumVar)	0.99	Functional Damage (DNA binding transcription factor and Zinc-ion binding activities are impacted)	155

## 4 Discussion

CHD remains one of the most common causes of non-infectious deaths in newborns [30]. Causative variants underlying CHD remain unknown in approximately 60 % of the cases [31]. In our study, we performed whole-exome sequencing to identify the underlying causative gene and found a pathogenic mutation, F184S, in GATA4. GATA4 is a zinc finger transcription factor located on chromosome 8p23.1-p22. It has six coding exons, comprises 442 amino acid proteins [32], and is highly expressed in cardiomyocytes at different developmental stages [14]. GATA4 contains two zinc finger domains, two transcriptional activation domains, and one nuclear location-signal domain [33]. Abnormalities in GATA4 during embryogenesis or in adult life cause structural and functional anomalies in the heart, such as ventricular septal defect (VSD), atrial septal defect (ASD), patent ductus arteriosus, endocardial cushion defect, tetralogy of Fallot, and pulmonary stenosis [34]; [35]. In addition, GATA4 forms complexes with other transcriptional factors, such as NKX2-5, SMAD1 and 4, and SRF [36].

The SwissModel server structural assessment and comparison tools indicated that the clash score and bad bonds were significantly increased in the GATA-4 F184S mutant homology structure. Furthermore, the impact of the F184 S missense mutation in GATA4 was evaluated using mutation analysis web tools such as missense 3D, PolyPhen-2, SIFT, Mutation Taster, and HOPE. PolyPhen-2 employs a supervised naïve Bayes classifier trained on annotations, conservation scores, and structural characteristics that describe amino acid substitutions. SIFT predicts whether an amino acid substitution affects protein function based on sequence homology and physical properties of amino acids. SIFT can be applied to naturally occurring non-synonymous polymorphisms and laboratory-induced missense mutations. MutationTaster predicts a variant to be *deleterious* or *benign*. The Grantham Matrix, an amino acid substitution matrix [37], was used to assess the physicochemical properties of amino acids and provides a score ranging from 0.0 to 215 based on the degree of difference between the wild-type amino acid (F) and the new amino acid (S) in GATA4.

HOPE is a web-based program that analyzes the structural consequences of point mutations in protein sequences [29]. The missense mutation F184S observed in GATA4 can interfere with the normal function of a cell and exert molecular effects by altering a protein's orthosteric or allosteric positions, interaction with substrates, or stability. HOPE predicted that the F184S missense mutation observed in a patient with CHD affects the binding of GATA4 with the DNA and the Zinc ions, thus potentially impairing the normal function of GATA4 transcription factors.

## 5 Conclusion

In conclusion, we identified a pathogenic variant of GATA4 that is responsible for CHD. This new variant may shed light on GATA4 function and its interactions with other proteins. Further studies using animal models are needed to identify the associated signalling pathways, better understand the disease, and search for potential gene therapy.

## Data availability

The data of this study is available from the corresponding author upon reasonable request.

## CRediT authorship contribution statement

**Mahmood Rasool:** Investigation, Data curation, Conceptualization. **Peter Natesan Pushparaj:** Software, Formal analysis. **Absarul Haque:** Writing – review & editing. **Ayat Mohammed Shorbaji:** Investigation. **Loubna Siraj Mira:** Validation, Data curation. **Sherin Bakhashab:** Supervision. **Mohamed Nabil Alama:** Data curation. **Muhammad Farooq:** Formal analysis. **Sajjad Karim:** Formal analysis. **Lars Allan Larsen:** Writing – review & editing, Supervision.

## Declaration of competing interest

The authors declare that they have no known competing financial interests or personal relationships that could have appeared to influence the work reported in this paper.

## References

[1] Aidinidou L., Chatzikyriakidou A., Giannopoulos A., Karpa V., Tzimou I., Aidinidou E., Fidani L. (2021 Jul 27). Association of *NFKB1, NKX2-5, GATA4* and *RANKL* gene polymorphisms with sporadic congenital heart disease in Greek patients. Balkan J. Med. Genet..

[2] Meller C.H., Grinenco S., Aiello H., Córdoba A., Sáenz-Tejeira M.M., Marantz P., Otaño L. (2020 Apr). Congenital heart disease, prenatal diagnosis and management. Arch. Argent. Pediatr..

[3] Miller J.L., De Veciana M., Turan S., Kush M. (2013). Firsttrimester detection of fetal anomalies in pregestational diabetes using nuchal translucency, ductus venosus Doppler, and maternal glycosylated hemoglobin. Am. J. Obstet. Gynecol..

[4] Panaitescu A.M., Nicolaides K. (2018). Maternal autoimmune disorders and fetal defects. J. Matern. Fetal Neonatal Med..

[5] Mandelbrot L. (2012). Fetal varicella - diagnosis, management, and outcome. Prenat. Diagn..

[6] Boucoiran I., Castillo E. (2018). No. 368-Rubella in pregnancy. J. Obstet. Gynaecol. Can..

[7] Prefumo F., Fichera A., Fratelli N., Sartori E. (2019 Jul). Fetal anemia: diagnosis and management. Best Pract. Res. Clin. Obstet. Gynaecol..

[8] Keighley C.L., Skrzypek H.J., Wilson A., Bonning M.A., Gilbert G.L. (2019 Aug). Infections in pregnancy. Med. J. Aust..

[9] Weston J., Bromley R., Jackson C.F., Adab N. (2016). Monotherapy treatment of epilepsy in pregnancy: congenital malformation outcomes in the child. Cochrane Database Syst. Rev..

[10] Tong Y.F. (2016). Mutations of NKX2.5 and GATA4 genes in the development of congenital heart disease. Gene.

[11] Zhang X., Wang J., Wang B., Chen S., Fu Q., Sun K. (2016 Jul 8). A novel missense mutation of GATA4 in a Chinese family with congenital heart disease. PLoS One.

[12] Williams K., Carson J., Lo C. (2019 Dec 16). Genetics of congenital heart disease. Biomolecules.

[13] Pikkarainen S., Tokola H., Kerkelä R., Ruskoaho H. (2004). GATA transcription factors in the developing and adult heart. Cardiovasc. Res..

[14] Li R.G., Xu Y.J., Wang J., Liu X.Y., Yuan F., Huang R.T., Xue S., Li L., Liu H., Li Y.J. (2018). GATA4 loss-of-function mutation and the congenitally bicuspid aortic valve. Am. J. Cardiol..

[15] Yang F., Zhou L., Wang Q., You X., Li Y., Zhao Y., Han X., Chang Z., He X., Cheng C., Wu C., Wang W.J., Hu F.Y., Zhao T., Li Y., Zhao M., Zheng G.Y., Dong J., Fan C., Yang J., Meng X., Zhang Y., Zhu X., Xiong J., Tian X.L., Cao H. (2014). NEXN inhibits GATA4 and leads to atrial septal defects in mice and humans. Cardiovasc. Res..

[16] Zhao R., Watt A.J., Battle M.A., Li J., Bondow B.J., Duncan S.A. (2008). Loss of both GATA4 and GATA6 blocks cardiac myocyte differentiation and results in acardia in mice. Dev. Biol..

[17] Jumper J., Evans R., Pritzel A., Green T., Figurnov M., Ronneberger O., Tunyasuvunakool K., Bates R., Žídek A., Potapenko A., Bridgland A., Meyer C., Kohl S.A.A., Ballard A.J., Cowie A., Romera-Paredes B., Nikolov S., Jain R., Adler J., Back T., Petersen S., Reiman D., Clancy E., Zielinski M., Steinegger M., Pacholska M., Berghammer T., Bodenstein S., Silver D., Vinyals O., Senior A.W., Kavukcuoglu K., Kohli P., Hassabis D. (2021 Aug). Highly accurate protein structure prediction with AlphaFold. Nature.

[18] Varadi M., Anyango S., Deshpande M., Nair S., Natassia C., Yordanova G., Yuan D., Stroe O., Wood G., Laydon A., Žídek A., Green T., Tunyasuvunakool K., Petersen S., Jumper J., Clancy E., Green R., Vora A., Lutfi M., Figurnov M., Cowie A., Hobbs N., Kohli P., Kleywegt G., Birney E., Hassabis D., Velankar S. (2022 Jan 7). AlphaFold Protein Structure Database: massively expanding the structural coverage of protein-sequence space with high-accuracy models. Nucleic Acids Res..

[19] Ittisoponpisan S., Islam S.A., Khanna T., Alhuzimi E., David A., Sternberg M.J.E. (2019). Can predicted protein 3D structures provide reliable insights into whether missense variants are disease associated?. J. Mol. Biol..

[20] Khanna T., Hanna G., Sternberg M.J.E., David A. (2021). Missense3D-DB web catalogue: an atom-based analysis and repository of 4M human protein-coding genetic variants. Hum. Genet..

[21] Ramachandran G.N., Ramakrishnan C., Sasisekharan V. (1963). Stereochemistry of polypeptide chain configurations. J. Mol. Biol..

[22] Ramachandran G.N., Sasisekharan V. (1968). Conformation of polypeptides and proteins. Adv. Protein Chem..

[23] Porter L.L., Rose G.D. (2011 Jan 4). Redrawing the Ramachandran plot after inclusion of hydrogen-bonding constraints. Proc. Natl. Acad. Sci. U. S. A..

[24] Chenn V.B., Arendall W.B., Headd J.J., Keedy D.A., Immormino R.M., Kapral G.J., Murray L.W., Richardson J.S., Richardson D.C. (2010). Acta Crystallogr..

[25] Benkert P., Biasini M., Schwede T. (2011). Toward the estimation of the absolute quality of individual protein structure models. Bioinformatics.

[26] Steinhaus R., Proft S., Schuelke M., Cooper D.N., Schwarz J.M., Seelow D. (2021 Jul 2). MutationTaster2021. Nucleic Acids Res..

[27] Adzhubei I.A., Schmidt S., Peshkin L., Ramensky V.E., Gerasimova A., Bork P., Kondrashov A.S., Sunyaev S.R. (2010). Nat. Methods.

[28] Sim N.L., Kumar P., Hu J., Henikoff S., Schneider G., Ng P.C. (2012 Jul). SIFT web server: predicting effects of amino acid substitutions on proteins. Nucleic Acids Res..

[29] Venselaar H., Te Beek T.A., Kuipers R.K., Hekkelman M.L., Vriend G. (2010 Nov 8). Protein structure analysis of mutations causing inheritable diseases. An e-Science approach with life scientist friendly interfaces. BMC Bioinf..

[30] Sadowski S.L. (2009). Congenital cardiac disease in the newborn infant: past, present, and future. Crit. Care Nurs. Clin..

[31] Zaidi S., Brueckner M. (2017). Genetics and genomics of congenital heart disease. Circ. Res..

[32] Soheili F., Jalili Z., Rahbar M., Khatooni Z., Mashayekhi A., Jafari H. (2018 Mar). Novel mutation of GATA4 gene in Kurdish population of Iran with nonsyndromic congenital heart septals defects. Congenit. Heart Dis..

[33] Chen J., Qi B., Zhao J., Liu W., Duan R., Zhang M. (2016). A novel mutation of GATA4 (K300T) associated with familial atrial septal defect. Gene.

[34] McCulley D.J., Black B.L. (2012). Transcription factor pathways and congenital heart disease. Curr. Top. Dev. Biol..

[35] Rajagopal S.K., Ma Q., Obler D., Shen J., Manichaikul A., Tomita-Mitchell A., Boardman K., Briggs C., Garg V., Srivastava D., Goldmuntz E., Broman K.W., Benson D.W., Smoot L.B., Pu W.T. (2007 Dec). Spectrum of heart disease associated with murine and human GATA4 mutation. J. Mol. Cell. Cardiol..

[36] Brody M.J., Cho E., Mysliwiec M.R., Kim T.G., Carlson C.D., Lee K.H., Lee Y. (2013). Lrrc10 is a novel cardiac-specific target gene of Nkx2-5 and GATA4. J. Mol. Cell. Cardiol..

[37] Grantham R. (1974). Amino acid difference formular to help explain protein evolution. Science.

